# Towards an Autonomous Vision-Based Unmanned Aerial System against Wildlife Poachers

**DOI:** 10.3390/s151229861

**Published:** 2015-12-12

**Authors:** Miguel A. Olivares-Mendez, Changhong Fu, Philippe Ludivig, Tegawendé F. Bissyandé, Somasundar Kannan, Maciej Zurad, Arun Annaiyan, Holger Voos, Pascual Campoy

**Affiliations:** 1 Interdisciplinary Centre for Security, Reliability and Trust, SnT - University of Luxembourg, 4 Rue Alphonse Weicker, L-2721 Luxembourg, Luxembourg; ludivig@hotmail.com (P.L.); tegawende.bissyande@uni.lu (T.F.B.); somasundar.kannan@uni.lu (S.K.); maciej.zurad@gmail.com (M.Z.); arun.annaiyan@uni.lu (A.A.); holger.voos@uni.lu (H.V.); 2 Centre for Automation and Robotics (CAR), Universidad Politécnica de Madrid (UPM-CSIC), Calle de José Gutiérrez Abascal 2, 28006 Madrid, Spain; fu.changhong@upm.es (C.F.); pascual.campoy@upm.es (P.C.)

**Keywords:** unmanned aerial vehicles, computer vision, animal tracking, face detection, vision-based control, object following, autonomous navigation, autonomous landing, anti-poaching

## Abstract

Poaching is an illegal activity that remains out of control in many countries. Based on the 2014 report of the United Nations and Interpol, the illegal trade of global wildlife and natural resources amounts to nearly $213 billion every year, which is even helping to fund armed conflicts. Poaching activities around the world are further pushing many animal species on the brink of extinction. Unfortunately, the traditional methods to fight against poachers are not enough, hence the new demands for more efficient approaches. In this context, the use of new technologies on sensors and algorithms, as well as aerial platforms is crucial to face the high increase of poaching activities in the last few years. Our work is focused on the use of vision sensors on UAVs for the detection and tracking of animals and poachers, as well as the use of such sensors to control quadrotors during autonomous vehicle following and autonomous landing.

## 1. Introduction

The use of unmanned aerial vehicles (UAVs) has increased rapidly in the last few decades and is now common in a variety of domains, ranging from leisure to rescue missions. UAVs have indeed become accessible to common consumers thanks to: the miniaturization of electronic components, including sensors, driven by other technologies, such as smart-phones; the increase in computational power for onboard CPUs; and the reduction in costs for this type of platform for robots. Thus, nowadays, UAVs are no longer solely reserved for military purposes. Several civilian applications (e.g., in agriculture, filming, *etc.*) have been developed recently. To accomplish the final take-off of UAV technology, many legal issues still need to be addressed for regulating the use of remotely-piloted or fully-autonomous UAVs. Nonetheless, there are specific scenarios where legal issues are irrelevant. These include areas of natural disasters or animal reserves where UAV technology can be essential in saving lives, either human or animal. Our current work focuses on the latter for supporting anti-poaching missions, which play an important role in protecting different species of animals around the world [1]. In Africa, animal poaching has reached critical levels due to the lack of resources on security and protection of the wildlife. The large size of national parks make it almost impossible to control different areas with the traditional surveillance methods with the limited number of security guards. In the necessary fight against poachers, reaction time is crucial. For example, it is estimated that a 10 minute delay is sufficient for killing and de-horning a rhino. Currently, the use of UAVs for anti-poaching activities is only limited to remotely-piloted systems with continuously-supervised images streamed from the onboard cameras [2]. In such approaches, however, expert UAV piloting knowledge is required. To maximize the usefulness of the UAVs, some autonomous capabilities must be included in the onboard systems. These capabilities must be designed to provide support in the surveillance of groups of animals, the recognition of poachers, as well as the tracking and following of their vehicles. In this work, we have focused on the development of these capabilities to define completely autonomous surveillance missions in the fight against poachers. In this work, an autonomous surveillance mission definition comprises the autonomous taking-off and following of a predefined position list (already available in almost all commercial UAVs systems), tracking animals, detecting poachers faces, tracking and following poachers vehicles and return, via an autonomously-landing scenario, on specific stations in order to recharge the batteries and prepare for the next surveillance flight.

Taking into account the large amount of security forces needed to cover the huge area (e.g. the Kruger National Park covers an area of 19,480 km${}^{2}$) of natural parks, we propose to equip all park security patrol vehicles with a UAV. These vehicles will have a landing/recharging platform on their roof (referred to as moving landing/charging stations). A set of static landing/charging stations should also be installed throughout the natural park area. These stations are needed to recover UAVs in cases where the closest security patrol vehicle is out of the range of a UAV’s battery endurance. Once the UAV takes off from one of these moving or static stations, it will follow a predefined patrolling trajectory. This trajectory could be modified autonomously in cases where an animal or a group of animals is detected. We assume that the onboard cameras are streaming the images to a security base or patrol vehicle in which a security guard can identify if an animal is hurt or under attack by poachers. The predefined trajectory may also be autonomously modified in case a group of poachers is detected. In this case, the system must detect the faces of the poachers to add them into a database of poachers. This database should be shared with international authorities in order to identify these individuals and have them prosecuted. Another case in which the UAV system should modify its trajectory is when a vehicle is detected. In this case, the system must be able to follow the vehicle in order to help the security authorities to pursue and catch potential poachers. Once the mission is completed or when the batteries of an UAV are about to be depleted, it will return to the closest moving or static landing/charging station and perform an autonomous landing. Based on the previously-established mission scenarios, this paper presents a number of vision-based algorithms. Some of these algorithms use aerial images for the tracking of animals, as well as for face detection. Other included algorithms use vision-based control systems in order to follow vehicles or to land UAVs autonomously on both moving and static platforms.

The remainder of this paper is organized as follows. Section 2 presents the related works regarding the computer vision techniques for visual tracking and face detection, the vision-based control approaches for UAVs and the anti-poaching activities with UAVs. Section 3 presents the related actions against poachers using UAVs. Section 4 shows the adaptive visual animal tracking approach. Section 6 presents the face detection approach for the identification of the poachers. Section 7 presents the vision-based control approach to control a quadrotor to follow vehicles and to accomplish autonomous landings on mobile platforms. Finally, Section 8 presents the conclusions and future works.

## 2. Related Works

### 2.1. Computer Vision Using Aerial Images

Similarly to other robotics platforms, depending on the application, UAVs may be set up with different sensor configurations. However, in the case of UAVs, the selection of the sensor configuration is more critical than in robotics platforms, such as ground vehicles, because of its limited payload. For this reason, the onboard sensors are required to present a wide working range, in order to be useful in different scenarios and for different purposes. Such requirements make the vision sensor the most auspicious sensor to be mounted onboard because of its low cost, high efficiency and its similarities to human vision. Numerous vision algorithms have already been developed to allow vision sensors to be used in a variety of applications. A vision sensor is commonly used for the detection and tracking of objects in images. It has been used this way for decades now with static cameras and on ground vehicles. More recently, such sensors started to be used on UAVs, as well. However, UAVs constitute a specific case where the complexity of vision tasks is substantially increased due to the rapid movements that such a platform can experience. These movements are not limited to lateral-forward, but also involve movements along the vertical axis, which affect the visioned size (*i.e.*, scale) of the tracked object. Therefore, well-tested vision algorithms which have been used for a long time on other platforms (e.g., on ground robots) are not as usable in UAV scenarios. They usually must be adapted to fit a particular use-case scenario, for example by including background subtraction capabilities. In previous research, one can find many object tracking surveys that show the alternative techniques and methodologies to follow [3,4,5]. Object tracking with a vision-based sensor relies on features present in the images. Those features can be detected based on color, edges, textures and optical flow. All of these tracking methods require an object detection mechanisms, as explained in detail by Yilmaz *et al.* [3].

Our work presents two different approaches of vision algorithms for specific purposes: the first one is related to object tracking, specifically animal tracking, while the second one is focused on face detection in order to identify and create a database of poachers’ identities. In the remainder of this sub-section, we will discuss research related to these specific computer vision approaches.

#### 2.1.1. Visual Tracking

Recently, visual tracking has been researched and developed fruitfully in the robot community. However, real-time robust visual tracking for arbitrary 3D animals (also referred to as visual animal model-free tracking), especially in UAV control and navigation applications, remains a challenging task due to significant animal appearance changes, variant illumination, partial animal occlusion, blur motion, rapid pose variation, cluttered background environments and onboard mechanical vibration, among others.

The typical visual tracking system should take into account three main requirements: (1) Adaptivity: this requires a reliable and sustained online adaptation mechanism to learn the real appearance of 3D animals; (2) Robustness: the tracking algorithm should be capable of following an animal accurately, even under challenging conditions; (3) Real time: this requires the tracking algorithm to process live images at a high frame rate and with an acceptable tracking performance, in order to generate consecutive and fast feedback vision estimations.

In the literature, visual tracking algorithms, based on the Lucas–Kanade optical flow, have been frequently utilized to track objects (e.g., [6] for UAVs). The 3D position of a UAV is estimated using a pre-defined reference object selected on the first image frame. However, this type of tracker cannot learn the animal appearance during tracking, and RANSAC [7] requires a large number of iterations (which implies heavy time consumption) to reach optimal estimation. Similarly, SIFT [8] and SURF [9] features have been used in visual tracking algorithms for object tracking. In summary, all such methods are known as feature-based visual tracking approaches.

The direct tracking method (*i.e.*, directly represent the object using the intensity information of all pixels in the image) was used to track objects from a UAV (*cf.* [10]). This type of tracker has been shown to perform better than the previously-mentioned and well-known feature-based algorithms. However, the direct tracking method also employs a fixed object template for the whole UAV tracking process. Although this type of tracker has been improved in [11] by manually adding many other templates, it still does not provide online self-taught learning. Moreover, the gradient descent method often falls into local minimum values and is relatively slow at achieving the global minimum.

An off-line learning algorithm for recognizing specified objects in UAV applications has been applied in [12] where a large amount of image training data is used to train off-line using a multi-layer perceptron artificial neural network (MLP-ANN). However, the object recognition is fixed/predefined instead of freewill objects, which are selected online. Besides the collection of the training image data, it is difficult to cover all of the challenging conditions that the UAV could encounter during an actual UAV flight. Furthermore, it is time consuming to empirically compute the optimal parameters for these kinds of off-line learning methods.

The adaptively-discriminative tracking method (referred to as the model-free tracking approach, where the tracked object is separated from its dynamic surrounding background using an adaptive binary classifier, which is updated with some positive and negative image samples) was applied in our previous work [13] and allowed for obtaining the accurate location of objects in an object tracking scenario from a UAV. Nevertheless, this tracker cannot provide the estimations of other motion model parameters, such as the rotation or scale information of the object. Even though incorporating these new parameter estimations into the tracker is straightforward, as declared by B. Babenko *et al.* [14] and tested in our different tracking experiments [15], the three performances mentioned above will dramatically decrease.

In our work, to handle the problems of drift, rapid pose variation and variant surrounding illumination, motivated by several works [16,17,18,19], the low-dimensional subspace representation scheme is applied as the practicable method to represent/model the 3D animal. The online incremental learning approach is utilized as the effective technique for learning/updating the appearance of a 3D animal. Moreover, the particle filter (PF) [20] and hierarchical tracking strategy are also employed to estimate the motion model of the 3D animal for UAV anti-poaching.

#### 2.1.2. Face Detection

A comprehensive survey on face detection algorithms has been published by Yang, Kriegman and Ahuja [21], where they list multiple approaches from the literature. We only discuss a few in the following:

One approach to face detection consists of looking for structural features that can be found in faces even when expression, viewpoint or lighting conditions change. An example of such an approach has been presented by Leung, Burl and Perona [22].

Another way to perform face detection is to make use of machine learning algorithms. As shown by Lanitis, Taylor and Cootes [23], it is possible to build machine learning classifiers to detect faces by training them with images of faces or specific features of faces. The resulting detection systems can than compare input images against learned patterns.

The third approach also makes use of machine learning algorithms, but in this case, the patterns that are used for the detection are not selected by the user, but by the machine learning algorithm itself. Examples of this approach are making use of a variety of machine learning algorithms, such as neural networks [24], hidden Markov models [25], support vector machines [26] or boosting [27]. This approach appears to be popular within the community due to the difficulty of reducing a face down to just a handful of features, especially when considering different lighting conditions and multiple viewing angles. Instead, machine learning algorithms manage to process a large number of training images in order to select a number of reliable features with a limited amount of human supervision.

For the purpose of our research, however, most of the presented methods are not suitable because of the real-time requirement of the face detection in our scenario. One commonly-used algorithm for real-time applications is the Viola and Jones boosting cascade algorithm [27] that we leverage in our work.

### 2.2. Vision as the Sensor for Control Applications in Robotics

Vision is a useful robotic sensor, since it mimics the human vision sense and allows one to extract non-contact measurements from the environment. The use of vision with robots has a long history, starting with the work of Shirai and Inoue [28], who describe how a visual feedback loop can be used to correct the position of a robot to increase the task accuracy. Today, vision systems are available from major vendors, and they are widely integrated in robotic systems. Typically, visual sensing and manipulation are combined in an open-loop fashion, “looking” and then “moving”. The accuracy of the results is directly dependent on the accuracy of the visual sensor, the robot end-effector and the controller. An alternative for increasing the overall accuracy of the system is to use a visual feedback control loop.

Visual servoing is no more than the use of vision at the lowest level, with simple image processing to provide reactive or reflexive behavior to servo position a robotic system. A classical visual servo control was developed for serial link robotic manipulators with the camera typically mounted on the end-effector, also called eye-in-hand. Even tough the first visual servoing systems had been presented by Sanderson [29] back in the 1980s, the development of visual control systems for robots has been fairly slow. However, many applications have appeared in the last two decades, due to the increase in computing power, which enables the analysis of images at a sufficient rate to “servo” a robotic manipulator.

Vision-based robot control using an eye-in-hand system can be classified into two groups: position-based and image-based visual servoing, PBVS and IBVS, respectively. PBVS involves the reconstruction of the target pose with respect to the robot and leads to a Cartesian motion planning problem. This kind of control is based on the three-dimensional information from the scene, so the geometric model of the object to track and a calibrated model of the camera are needed. Then, the estimation of the position and orientation of the object is obtained. The PBVS design is sensitive to the camera calibration, which is particularly challenging when using a low quality camera. In contrast, for IBVS, the control task is defined in terms of image features. A controller is designed to maneuver the image features to a desired configuration. The original Cartesian motion planning problem is solved. The approach is inherently robust to camera calibration and target modeling errors which in turn reduces the computational cost. However, this configuration implies an extra complexity for the control design problem.

### 2.3. Vision-Based UAV Control

There are many visual servoing applications for UAVs present in the literature. Different vision-based algorithms have been used to follow a car from a UAV [30,31,32]. Visual terrain following (TF) methods have been developed for a vertical take-off and landing (VTOL) UAVs [33]. In [34], a description of a vision-based algorithm to follow and land on a moving platform and other related tasks are proposed. A cooperative strategy has been presented in [35] for multiple UAVs to pursue a moving target in an adversarial environment. The low-altitude road-following problem for UAVs using computer vision technology was addressed in [36]. The people-following method with the parallel tracking and mapping (PTAM) algorithm has been developed in [37]. Contrary to the above discussed research, the autonomous target following and landing approach presented in this work is based on the control of the lateral, longitudinal, vertical and heading velocities of the quadrotor to modify its position to follow and land on a predefined platform.

Related to the autonomous landing, there exists previous work that is focused on the theoretical control part of this problem, which has been examined in simulated environments, such as [38]. This presents a classical PID control using the SIFT vision algorithm, proving the feasibility of this algorithm for this specific task and testing the controllers in a simulated environment. In [39], the authors have evaluated the use of visual information at different stages of a UAV control system, including a visual controller and a pose estimation for autonomous landing using a chessboard pattern. In [40], a visual system is used to detect, identify a landing zone (helipad) and confirm the landing direction of the vehicle. The work in [41,42] proposed an experimental method of autonomous landing on a moving target, by tracking a known helipad and using it to complement the controller IMU + GPS state estimation. Other research has also been able to demonstrate autonomous landing with a VTOL aircraft [43]. This research makes use of a fusion sensor control system using GPS to localize the landmark, vision to track it and sonar for the last three meters of the autonomous landing task.

The work in [44,45] used a method to fuse visual and inertial information in order to control an autonomous helicopter landing on known landmarks. In [46], the authors presented the results of a fusion sensor of GPS, compass and vision with a PID controller to track and follow the landing location and land on a landmark. Overall, all of the aforementioned works are related to fixed wing aircraft or helicopters.

Nowadays, the increasing popularity of multi-copters (commonly quadcopters) calls the attention of the research to this topic. Some examples include work presented by Lange in [47] where a visual system is used to estimate a vehicle position relative to a landing place. In [48,49], a decomposition of a quadrotor control system to an outer-loop velocity control and an inner-loop attitude control system is proposed. In this work, the landing controller consists of a linear altitude controller and a nonlinear 2D-tracking controller. The work in [50] shows a deep theoretical work of a non-linear controller of a quadrotor that is built on homography-based techniques and Lyapunov design methods. Recently, [51,52] have shown two different methods to be used with micro-UAVs for autonomous takeoff, tracking and landing on a moving platform. This work is based on optical flow and IR landmarks to estimate the aircraft’s position. The work in [53] displays the experiments of the autonomous landing of an AR.Drone on a landing pad mounted on top of a kayak. A deep review of the different control techniques for autonomous navigation, guidance and control for UAVs is presented in [54,55,56,57].

## 3. Related Actions against Poachers

To the best of our knowledge, the only working approach that relies on UAVs for fighting against poaching is the initiative conducted by SPOTS-Air Rangers [2]. As is mentioned on their web page, they are a “registered Section 21 Conservation Company focused purely on the conservation and the protection of any and all threatened species”. The company is using UAVs in Africa, specifically South Africa, for the specific task of poacher detection. They use fixed-wing aircraft equipped with a high quality thermal camera, among other sensors needed to fly. The aircraft is provided by Shadowview, which is also a non-profit organization providing multiple UAS solutions for conservation and civilian projects [58]. The main goal of Air Rangers is to participate in the fight against poaching, through the detection of potential poachers. They also use UAVs to track animals and thus improve the wildlife census system. Furthermore, this company leveraged their aircraft for burn assessment and biomass management. Based on what we could extract from SPOTS’s website, they are not using any software for autonomous detection of poaching situations, by, e.g., detecting poachers, vehicles, camps and strange behaviors. No specific algorithms to increase the effectiveness of the patrol trajectories are shown in their tasks descriptions either.

We claim that the work of this company and its project in Africa could be improved with the automation techniques that are discussed in our work [1] and whose design and implementation are presented in this paper. Our objective is to increase the number of detected poachers and to improve the effectiveness and efficiency of autonomous flights. It is noteworthy that similar initiatives are currently in the process of being applied. Those include the Air Sheppard project by the Lindberg Foundation and the wildlife conservation UAV challenge organized by Princess Aliyah Pandolfi, as well as the Kashmir World Foundation [59], in which more than 50 teams are involved.

## 4. Adaptive Visual Animal Tracking Using Aerial Images

In this section, the details of the presented adaptive visual animal tracking onboard a UAV for anti-poaching are introduced. This visual algorithm can overcome the problems generated by the various challenging situations, such as significant appearance change, variant surrounding illumination, partial animal occlusion, rapid pose variation and onboard mechanical vibration.

### 4.1. Adaptive Visual Animal Tracking Algorithm

**Figure 1:**
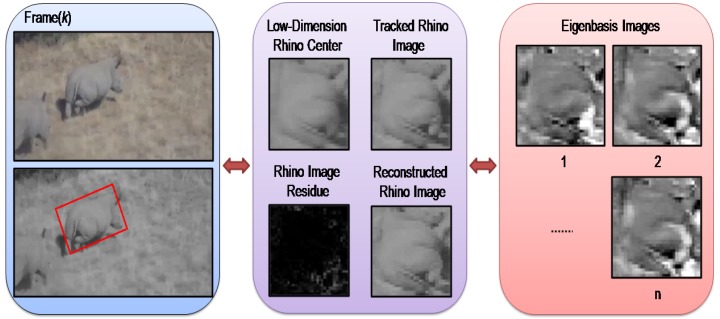
The PCA subspace-based tracking of a 3D rhino in our work, where each rhino image is re-sized to 32 × 32 pixels, and the reconstructed rhino image is constructed using the eigenbasis. Moreover, the eigenbasis images are sorted based on their according eigenvalues.

Online incremental subspace learning methods, e.g., by G. Li *et al.* [60], T. Wang *et al.* [61], D. Wang *et al.* [62] and W. Hu *et al.* [63], have obtained promising tracking performances. Recently, D. Ross *et al.* [64] have presented an online incremental learning approach for effectively modeling and updating the tracking of objects with a low dimensional principal component analysis (PCA) subspace representation method, which demonstrated that PCA subspace representation with online incremental updating is robust to appearance changes caused by rapid pose variation, variant surrounding illumination and partial target occlusion, as expressed by Equation (1) and shown in Figure 1. In addition, PCA has also been demonstrated in [19,65] to have those advantages in tracking applications. A. Levey *et al.* [66] and P. Hall *et al.* [67] have done works similar to those in [64], although [66] did not consider the changing of the subspace mean when new data arrive, while the forgetting factor is not integrated in [67], which generates a higher computational cost during the tracking process.

(1) O=Uc+e

In Equation (1), O represents an observation vector, c indicates the target coding coefficient vector, U denotes the matrix of column basis vectors and e is the error term, which is the Gaussian distribution with small variances.

The main procedures of the online incremental PCA subspace learning algorithm with subspace mean updating [64] are as follows: Given a set of training images $S_{a}=\left\{S_{1},S_{2},\ldots ,S_{n}\right\}\in R^{d\times n}$, the appearance model of the 3D animal can be computed by the singular value decomposition (SVD) of the centered data matrix $\left[\left(S_{1}-{\bar{S}}_{a}\right)\cdots \left(S_{n}-{\bar{S}}_{a}\right)\right]$, denoted by ($S_{1}-{\bar{S}}_{a}$), *i.e.*, $\left(S_{a}-{\bar{S}}_{a}\right)=U\Sigma V^{⊤}$, where ${\bar{S}}_{a}=\frac{1}{n}\sum_{i=1}^{n}S_{i}$ is the sample mean of the training images.

If a new set of images $S_{b}=\left\{S_{n+1},S_{n+2},\ldots ,S_{n+m}\right\}\in R^{d\times m}$ arrives, then the mean vectors of $S_{b}$ and $S_{c}=\left[S_{a}\;S_{b}\right]$ are computed, *i.e.*, ${\bar{S}}_{b}=\frac{1}{m}\sum_{i=n+1}^{n+m}S_{i}$, ${\bar{S}}_{c}=\frac{n}{n+m}{\bar{S}}_{a}+\frac{m}{n+m}{\bar{S}}_{b}$. Because the SVD of $\left(S_{c}-{\bar{S}}_{c}\right)$ is equal to the SVD of concatenation of $\left(S_{a}-{\bar{S}}_{a}\right)$, $\left(S_{b}-{\bar{S}}_{b}\right)$ and $\sqrt{\frac{nm}{n+m}}\left({\bar{S}}_{a}-{\bar{S}}_{b}\right)$, which is denoted as $\left(S_{c}-{\bar{S}}_{c}\right)=U^{\prime }\Sigma^{\prime }V^{\prime ⊤}$, this can be done efficiently by the R-SVD algorithm, *i.e.*:

(2) $$U^{\prime }=\left[U\;\tilde{E}\right]\tilde{U},\;\Sigma^{\prime }=\tilde{\Sigma }$$

where, $\tilde{U}$ and $\tilde{\Sigma }$ are calculated from the SVD of *R*: $\left[\begin{array}{cc} \Sigma & U^{⊤}E\\ 0 & \tilde{E}\left(E-UU^{⊤}E\right) \end{array}\right]$, *E* is the concatenation of $\left(S_{b}-{\bar{S}}_{b}\right)$ and $\sqrt{\frac{nm}{n+m}}\left({\bar{S}}_{a}-{\bar{S}}_{b}\right)$, $\tilde{E}$ represents the orthogonalization of $E-UU^{⊤}E$ and *U* and Σ are the SVD of $\left(S_{a}-{\bar{S}}_{a}\right)$.

Taking the forgetting factor, *i.e.*, θ∈(0,1], into account for balancing between previous and current observations to reduce the storage and computation requirements, the *R* and ${\bar{S}}_{c}$ are modified as below:

(3) $$R=\left[\begin{array}{cc} \eta \Sigma & U^{⊤}E\\ 0 & \tilde{E}\left(E-UU^{⊤}E\right) \end{array}\right]$$

(4) $${\bar{S}}_{c}=\frac{\eta n}{\eta n+m}{\bar{S}}_{a}+\frac{m}{\eta n+m}{\bar{S}}_{b}$$

where θ=1 means that all previous data are included to adapt to the changing appearance of the 3D animal.

For the visual animal tracking task of the UAV, it can be formulated as an inference problem with a Markov model and hidden state variables. Given a set of observed images $O_{k}=\left\{O_{1},O_{2},\ldots ,O_{k}\right\}$ at the *k*-th frame, the hidden state variable $X_{k}$ can be estimated as below:

(5) $$\begin{array}{c} p\left(X_{k}|O_{k}\right)\propto p\left(O_{k}|X_{k}\right)\cdot \;\\ \;\int p\left(X_{k}|X_{k-1}\right)p\left(X_{k-1}|O_{k-1}\right)dX_{k-1} \end{array}$$

where $p(X_{k}|X_{k-1})$ is the dynamic (motion) model between two consecutive states and $p(O_{k}|X_{k})$ represents the observation model that estimates the likelihood of observing $O_{k}$ at the state $X_{k}$. The optimal state of the tracking animal given all of the observations up to the *k*-th frame is obtained by the maximum *a posteriori* estimation over N samples at time *k* by:

(6) $${\hat{X}}_{k}=\arg\max_{X_{k}^{i}}p\left(O_{k}^{i}|X_{k}^{i}\right)p\left(X_{k}^{i}|X_{k-1}\right),i=1,2,\ldots ,N$$

where $X_{k}^{i}$ is the *i*-th sample of the state $X_{k}$ and $O_{k}^{i}$ denotes the image patch predicted by $X_{k}^{i}$.

#### 4.1.1. Dynamic Model

**Figure 2:**
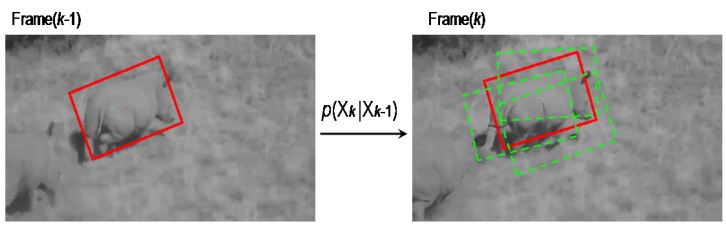
The dynamic model of visual rhino tracking.

In this application, we aim to use four parameters for constructing the motion model $X_{k}$ of the 3D animal to close the vision control loop: (I) location *x* and *y*; (II) scale factor *s*; (III) rotation angle *θ* of the 3D animal in the image plane, *i.e.*, $X_{k}=\left(x_{k},y_{k},s_{k},\theta_{k}\right)$, which can be modeled between two consecutive frames; it is called similarity transformation in [68], as shown in the Figure 2. The state transition is formulated by a random walk:

(7) $$p\left(X_{k}|X_{k-1}\right)=N\left(X_{k};X_{k-1},\Psi \right)$$

In Equation (7), Ψ is the diagonal covariance matrix, *i.e.*, $\Psi =(\sigma_{x}^{2},\sigma_{y}^{2},\sigma_{s}^{2},\sigma_{\theta }^{2})$. However, the efficiency (*i.e*., how many particles should be generated) and effectiveness (*i.e.*, how well particle filter should approximate the *a posteriori* distribution, which depends on the values in **Ψ**) of the PF should be a trade off. Larger values in **Ψ** and more particles will obtain higher accuracy, but at the cost of more storage and computation expenses. We solved this problem in Section 4.2.

#### 4.1.2. Observation Model

In this work, we apply the low-dimensional PCA subspace representation to describe the tracked 3D animal. Thus, a probabilistic interpretation of PCA should be modeled for the image observations. The probability is inversely proportional to the distance from the sample to the reference point (*i.e.*, center) of the subspace, which includes two types of distances: (i) the distance-to-subspace, $d_{to}$; (ii) the distance-within-subspace, $d_{within}$.

The probability of $d_{to}$ is defined as:

(8) $$p_{d_{to}}\left(O_{k}|X_{k}\right)=N\left(O_{k};\mu ,UU^{⊤}+\varepsilon I\right)$$

where ***μ*** is the center of the subspace, I represents the identity matrix and εI denotes the Gaussian noise.

(9) $$p_{d_{within}}\left(O_{k}|X_{k}\right)=N\left(O_{k};\mu ,U\Sigma^{-2}U^{⊤}\right)$$

where Σ represents the matrix of singular values corresponding to the columns of U.

Hence, the probability of the observation model is as follows:

(10) $$\begin{array}{ccc} p(O_{k}|X_{k}) & = & p_{d_{t}}\left(O_{k}|X_{k}\right)p_{d_{w}}\left(O_{k}|X_{k}\right)\\ & = & N\left(O_{k};\mu ,U\Sigma^{-2}U^{⊤}\right)N\left(O_{k};\mu ,U\Sigma^{-2}U^{⊤}\right) \end{array}$$

Moreover, the robust error norm, *i.e.*, $\rho \left(x,y\right)=\frac{x^{2}}{x^{2}+y^{2}}$, rather than the quadratic error norm, has been applied to reduce the noise effects.

### 4.2. Hierarchy Tracking Strategy

**Figure 3:**
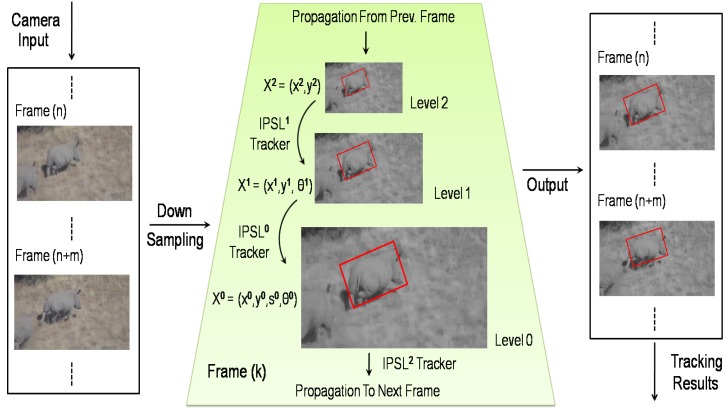
Our adaptive visual tracker for 3D animal tracking. The *k*-th frame is downsampled to create the multi-resolution structure (middle). In the motion model propagation, lower resolution textures are also initially used to reject the majority of samples at relatively low cost, leaving a relatively small number of samples to be processed at higher resolutions. The IPSL${}^{p}$ represents the incremental PCA subspace learning-based (IPSL) tracker in the *p*-th level of the pyramid.

In the visual animal tracking application of UAVs, we find that an incremental PCA subspace learning-based (IPSL) visual tracker is sensitive to large displacements or strong motions. Although the value in **Ψ** (in Equation 7) can be set to be larger and more particles can be generated to get more tolerance for these problems, more noises will be incorporated from those particles, however, and the requirements of storage and the computation cost will be higher, which influences the real-time and accuracy performances. Therefore, the hierarchical tracking strategy, based on the multi-resolution structure, has been proposed in our work to deal with these problems, as shown in Figure 3. Nevertheless, there must be a compromise between the number of levels required to overcome the large inter-frame motion and the amount of visual information required to update the appearance of a 3D animal for estimating the motions. The main configurations for hierarchical visual animal tracking are as follows.

### 4.3. Hierarchical Structure

Considering that the image frames are down-sampled by a ratio factor of two, the number of pyramid levels ($N_{PL}$) of the multi-resolution structure is defined as a function below:

(11) $$N_{PL}=\left\lfloor log_{2}\frac{min\{T_{W},T_{H}\}}{minSizes}\right\rfloor$$

where ⌊*⌋ is the largest integer not greater than value *, $T_{W}$ and $T_{H}$ represent the width and height of animal T in the highest resolution image (*i.e.*, the highest level of pyramid: Level 0), respectively. Additionally, minSizes is the minimum size of the animal in the lowest resolution image (*i.e*., the lowest-level of pyramid: $p_{min}$ level, $p_{min}=N_{PL}$-1), in order to have enough information to estimate the motion model at that level. Thus, if the minSizes is set in advance, the $N_{PL}$ directly depends on the width/height of tracking animal T. In this application, the number of pyramid levels is $N_{PL}=3$; then, *p* is initialized as p={2,1,0}.

### 4.4. Particle Filter Setup

Since the multi-resolution structure provides the computational advantage to analyze textures and update the appearance model in low resolution images and the lowest resolution image is good for estimating the location of tracking the animal, with the increase of resolution, more details from visual information can be used to estimate more parameters in the motion model. In this work, the motion models estimated in different resolution frames are defined as follows based on [68]:

Level 2:

$\;X_{k}^{2}=\left(x_{k}^{2},y_{k}^{2}\right)$, *i.e.*, translation

Level 1:

$\;X_{k}^{1}=\left(x_{k}^{1},y_{k}^{1},\theta_{k}^{1}\right)$, *i.e.*, translation+rotation

Level 0:

$\;X_{k}^{0}=\left(x_{k}^{0},y_{k}^{0},s_{k}^{0},\theta_{k}^{0}\right)$, *i.e.*, similarity

### 4.5. Motion Model Propagation

Taking into account the fact that the motion model estimated at each level is used as the initial estimation of motion for the next higher resolution image, the motion model propagation is defined as follows:

$$x_{k}^{p-1}=2x_{k}^{p},\;y_{k}^{p-1}=2y_{k}^{p}$$

(12) $$\theta_{k}^{p-1}=\theta_{k}^{p}$$

$$s_{k}^{p-1}=s_{k}^{p}$$

where *p* represents the *p*-th level of the pyramid, $p=\left\{p_{min},p_{min}-1,\ldots ,0\right\}=\left\{N_{PL}-1,N_{PL}-2,\ldots ,0\right\}$, and *k* is the *k*-th frame.

After finding the motion model in the *k*-th frame, this motion model is sent as the initial estimation to the highest pyramid level of the (*k*+1)-th frame, as shown in Figure 3:

$$x_{k+1}^{p_{min}}=\frac{x_{k}^{0}}{2^{p_{min}}},\;y_{k+1}^{p_{min}}=\frac{y_{k}^{0}}{2^{p_{min}}}$$

(13) $$\theta_{k+1}^{p_{min}}=\theta_{k}^{0}$$

$$s_{k+1}^{p_{min}}=s_{k}^{0}$$

Besides the propagation of motion models, the majority of particles will be rejected based on their particle weights in the lower resolution image. In other words, it is not necessary to generate a larger number of samples to estimate the same parameters in the higher resolution image, leaving a higher tracking speed and better accuracy than a single full-resolution-based voting process. The reject particle number is defined as:

(14) $$N_{R}^{p}=\alpha^{p}N_{P}^{p}$$

where $\alpha^{p}$ is the reject ratio ($0<\alpha^{p}<1$) at the *p*-th level in the pyramid and $N_{P}^{p}$ is the number of generated particles.

In the rejected particles, the particle with maximum weight is called the critical particle ($C_{k}^{p}$). Taking the *x* position for example, the distance between *x* of $C_{k}^{p}$ and $x_{k}^{p}$ is denoted as the heuristic distance ($H_{k}^{p}$). Therefore, for the searching range propagation, *i.e.*, $\sigma_{x}$, it is defined as:

(15) $$\sigma_{\left(k,x\right)}^{p-1}=2H_{k}^{p}$$

where $\sigma_{\left(k,x\right)}^{p-1}$ is the variance of *x* translation at the (p-1)-th level of the pyramid of the *k*-th frame. Additionally, the other motion model parameters have similar propagations during the animal tracking process.

### 4.6. Block Size Recursion

The multi-block size adapting method has been used to update the 3D animal with different frequencies, *i.e.*, a smaller block size means more frequent updates, making it faster for modeling appearance changes. Because the image at the lowest level of the pyramid has less texture information, thus the recursion of block size ($N_{B}$) is given as below:

(16) $$N_{B}^{p-1}=\left\lfloor \frac{N_{B}^{p}}{log_{2}\left(1+p\right)}\right\rfloor$$

where ⌊*⌋ is the largest integer not greater than value *, *p* represents the *p*-th level in the pyramid and *k* is the *k*-th frame.

All of the approaches introduced in this section are integrated to ensure higher accuracy and the real-time performance of the 3D animal tracking from UAVs. The real tests are discussed in the section below.

## 5. Visual Animal Tracking Evaluation and Discussion

This section discusses the 3D animal tracking performance of our presented algorithm, which is evaluated with the ground truth datasets generated from real UAV flight tests in Africa. In the different experiments, we have used the Robot Operating System (ROS) framework to manage and process image data.

### 5.1. Ground Truth Generation

**Figure 4:**
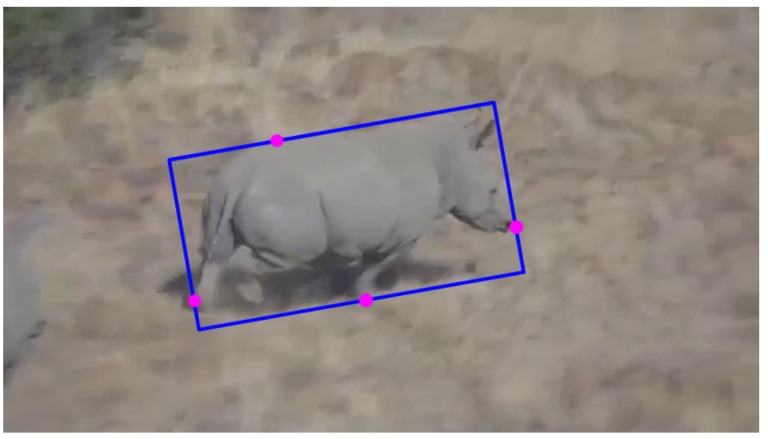
Reference rectangle of the ground truth. The reference rectangle has included all of the pixels of the tracked animal, and the pink points are key pixels for locating the reference rectangle.

Manually-generated ground truth databases have been leveraged to analyze the performance of our visual animal tracker. Figure 4 shows a reference rectangle for generating the ground truth. The center location, rotation, width and height of the tracked animal can be obtained frame-to-frame based on the reference rectangle.

### 5.2. Real Test of Visual Animal Tracking

**Figure 5:**
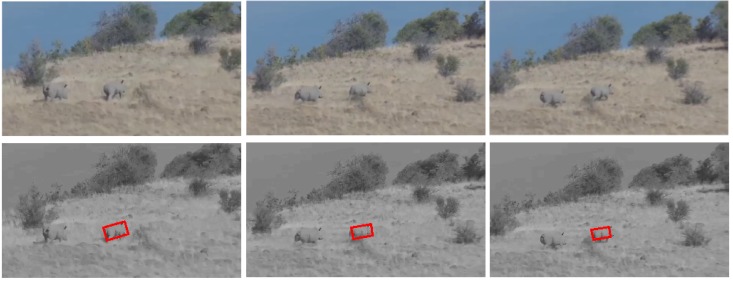
Visual rhino tracking. The red rectangle shows the estimated location of the running rhino.

**Figure 6:**
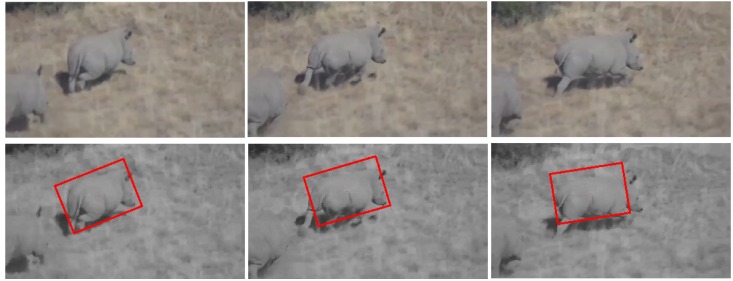
Visual rhino tracking.

**Figure 7:**
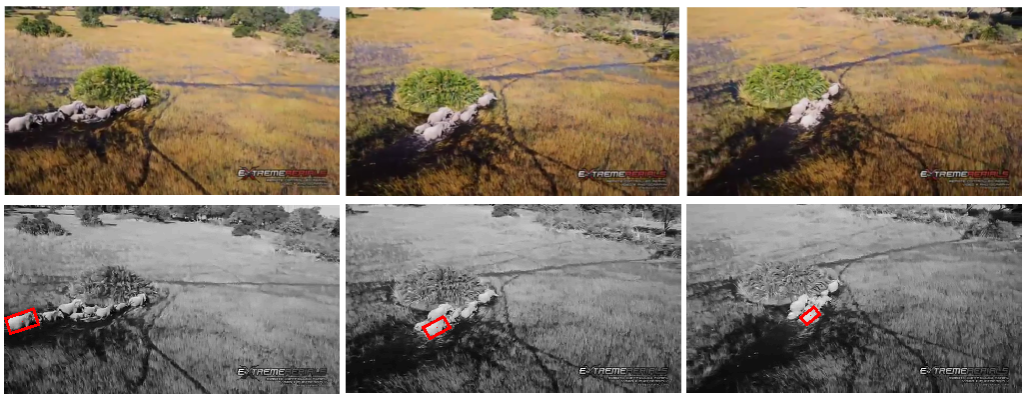
Visual elephant tracking.

**Figure 8:**
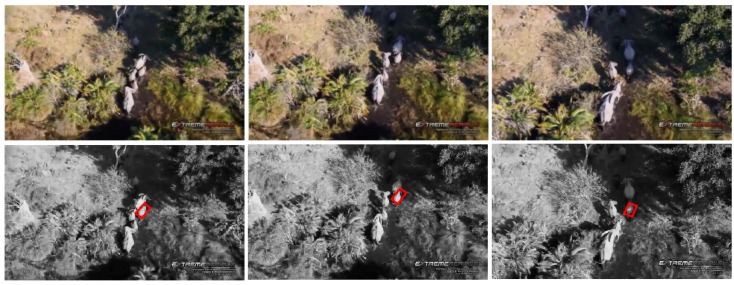
Visual elephant tracking.

In the first experimental settings, we have selected the rhino and elephant as typical threatened animal species in Africa to test our proposed visual tracking algorithm. Some tracking results are shown in Figure 5, Figure 6, Figure 7 and Figure 8.

#### 5.2.1. Test 1: Rhino Tracking

In this test, the visual rhino tracking estimation contains three main challenging factors: (I) 3D appearance change (e.g., different views from the onboard camera and random running of the rhino); (II) partial rhino occlusion (e.g., occluded by bushes); (III) protective coloration (*i.e.*, the color of the rhino body is similar to the background). The tracking results, as illustrated by some randomly-selected examples in Figure 5, show that our presented visual tracker can locate the running rhino (*i.e.*, the front (right) rhino with the red rectangle) accurately from a flying UAV, even in in face of varying running speeds. Although those two running rhinos have extremely similar appearances, our visual tracker has not been confused to then track the front (right) rhino stably. The center location error is defined as the Euclidean distance from the tracked animal center to the ground truth center at each image frame. The average errors of the center location, rotation angle, width and height are three pixels, two degrees, three pixels and two pixels, respectively. The performance of tracking the back (left) rhino also displays similar results.

#### 5.2.2. Test 2: Rhino Tracking

Compared to Test 1, the flying UAV is closer to the running rhinos, as shown in Figure 6. The vision-based rhino tracking estimation now contains three main challenging factors: (I) 3D appearance change; (II) rapid pose variation; (III) partial rhino occlusion. In this test, the real-time and adaptive performances of our presented visual tracker have guaranteed the accuracy of the location for this visual animal tracking application. The average errors of the center location, rotation angle, width and height are 11 pixels, four degrees, seven pixels and four pixels, respectively.

#### 5.2.3. Test 3: Elephant Tracking

In this test, our presented visual tracker has been used to locate an elephant, by trying to track one moving elephant in a group of moving elephants, as shown in Figure 7. The visual tracking estimation contains three main challenging factors: (I) 3D appearance change; (II) clustered tracking background; (III) partial elephant occlusion. The average errors of the center location, rotation angle, width and height are three pixels, three degrees, two pixels and four pixels, respectively.

#### 5.2.4. Test 4: Elephant Tracking

In this test, the tracking object is the same as the one in Test 3, *i.e.*, moving elephant, although with different challenges. The vision-based tracking estimation contains three main challenging factors: (I) 3D appearance change; (II) clustered tracking background; (III) illumination variation; as the shadow areas shown in the right-top corner of the images in Figure 8. The average error of the center location, rotation angle, width and height are three pixels, two degrees, four pixels and two pixels, respectively.

In general, the vision estimations are well-matched with the real location (ground truth) of animals in all of the visual animal tracking tests, without many salient outliers.

The videos related to some of the experiments presented in this section are available on a website [69].

## 6. Face Detection Using Aerial Images for Poachers’ Detection and Identification

In this section, we detail our work on face detection for identifying poachers. The information acquired may be used for a number of purposes, including future UAV control applications to follow poachers, as well as to create and maintain a database of poachers, which can be distributed to the competent authorities. Our work leverages existing face detections systems and implements them for UAV platforms. While similar research involving face recognition on UAVs [70] and face detection for UAVs [71] or for robotic platforms [72] has been investigated previously, these works cover very specific scenarios where the subjects are relatively close to the drone while flying in controlled indoor environments. There is still a lack of research regarding real-time face detection for more real-world outdoor scenarios.

### 6.1. Face Detection Approach

To satisfy real-time requirements in face detection, we are using a commonly-applied face detection algorithm, which was presented by Viola and Jones [27]. The boosting cascade algorithm has three main characteristics:

- It is easy to calculate integral features
- It uses machine learning using AdaBoost
- It leverages the cascade system for speed optimization

#### 6.1.1. Feature Detection

**Figure 9:**
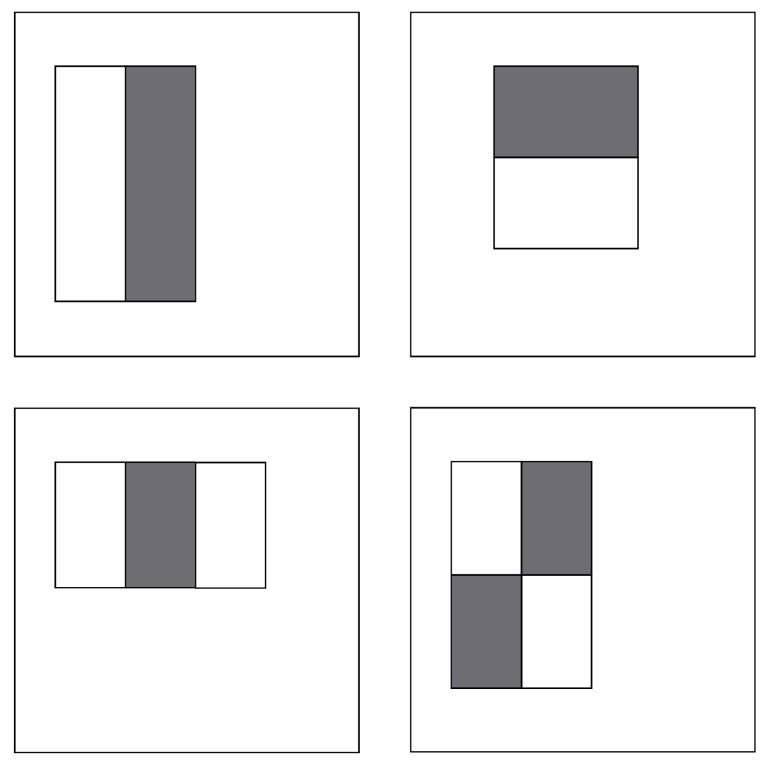
Integral image features used in boosting cascade face detections.

**Figure 10:**
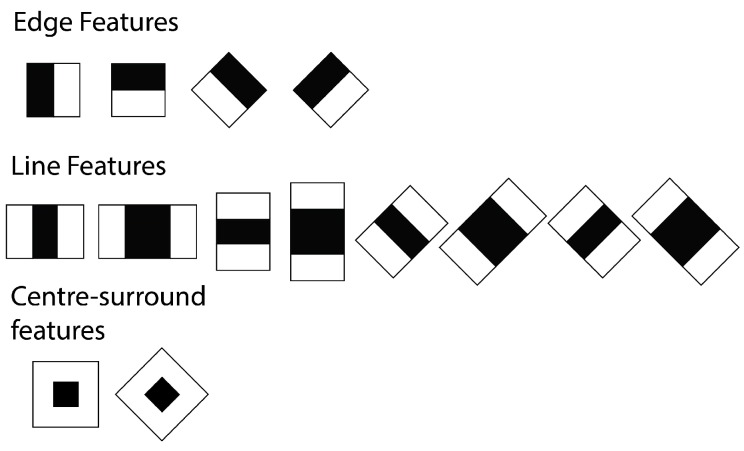
Total set of features used by the OpenCV detection.

For faster feature detection, the system avoids using pixel-based features. Instead, it makes use of Haar-like features [73], where the system checks the sums of rectangular areas against the sums of other rectangular areas. There are four types of integral image features, which can be seen in Figure 9. For the detection, the pixels within the white rectangles are added together and then subtracted by the sum of all of the pixels within the grey area. Since the algorithm only works on grey-scale images, there is only one value for each pixel. This type of feature can be calculated with a single pass over the image. In addition to this, the implementation that we used applies an additional extension [74] for the Haar-Like features, as seen in Figure 10. This extension allows for features that are rotated at a 45° angle, as well as center-surround features, which define a point feature rather than an edge or a line.

**Figure 11:**
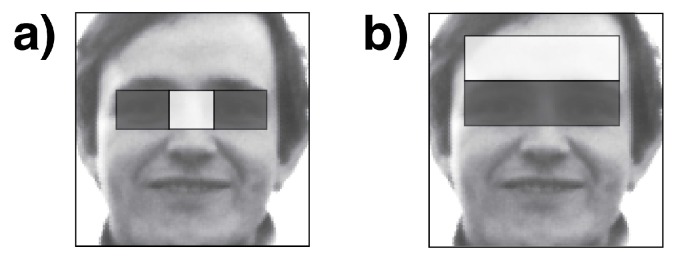
Example of features. (**a**) the nose-bridge tends to be brighter than the eyes; (**b**) the forehead being brighter than the eye region below.

In Figure 11, one can see two features selected by Adaboost. Feature (a) capitalizes on the fact that the nose-bridge tends to be brighter than the eyes. Feature (b) works with the forehead being brighter than the eye region below.

#### 6.1.2. AdaBoost

The detection of the features is performed by a machine learning algorithm known as AdaBoost [75] (adaptive boosting). In principle, many weak classifiers are needed to produce one much more accurate strong classifier. In our case, this system is ideal for the integral features, since the initial weak classifiers only have to be better than random. Adaboost requires an initial learning period with labeled and carefully cropped training data.

#### 6.1.3. Cascade System

To reduce the processing time of a large number of features, we use a cascade system where the most defining features are tested first. This aims to solve the main problem of the AdaBoost system where a large number of features are required, due to the very basic nature of the Haar-Like features. The cascade approach allows the algorithm to discard many false detections without having to check all of the features in the system. The order in which the features are being tested may significantly speed up the face detection. To put this into perspective, the test system from [27] had 6000 features, which were split into 38 cascade stages. The use of the cascade system resulted in a 15-fold speed increase. The downside to using this method is an additional training period, which is used to determine in which order the features are being applied. The boosting cascade system may have long training phases by an “order of weeks” ([76], p. 583).

### 6.2. Implementation

For the purpose of our research, the existing OpenCV cascade classifier implementation has been applied. The version used is OpenCV 2.4.8 since it comes with ROS Indigo and because there is an existing CV_bridge which translates the ROS images topic into OpenCV Mat images. It is important to know that the classifiers need to be trained beforehand. Fortunately, OpenCV comes with a set of pre-trained models, which are perfectly suited for face detection. The next step of the detection is to prepare the images for the detection. The classifier requires the color images to be transformed into a grey-scale color space. For improved detection, depending on the lighting conditions, either additional contrast or a histogram equalization is applied. Optionally, the may also be scaled at this stage. With respect to larger resolution images, this is the best way to improve performance. In this case, however, this is not ideal, since it reduces the detection of people that are further away from the camera. This is partially due to the wide angle lens of the camera, which makes people appear smaller in images. One should also keep in mind that people tend to keep their distance from UAVs.

At the detection level, there are a number of settings that may be used to improve the number of correct detections and reduce the number of false detections:

- *Cascade*: OpenCV comes with a number of different face detection cascade systems. For this research, haarcascade_frontalface_alt_tree.xml has been selected.
- *scaleFactor*= 1.2: The algorithm scans the input image in multiple iterations, each time increasing the detection window by a scaling factor. A smaller scaling factor increases the number of detections, but also increases the processing time.
- *minNeighbors* = 3: This refers to the number of times a face needs to be detected before it is accepted. Higher values reduce the number of false detections.
- *Flags:* There a number of additional flags in existence. In this particular case, the flag CV_HAAR_DO_CANNY_PRUNING is used because it helps to reduce the number of false detections and it also improves the speed of the detection.
- *minSize* and *maxSize*: These parameters limit the size of the search window. With fixed cameras, this is beneficial to improve speed, but in this case, these values are set very broadly because the distance from the subjects is constantly changing.

### 6.3. Drone Setup

**Figure 12:**
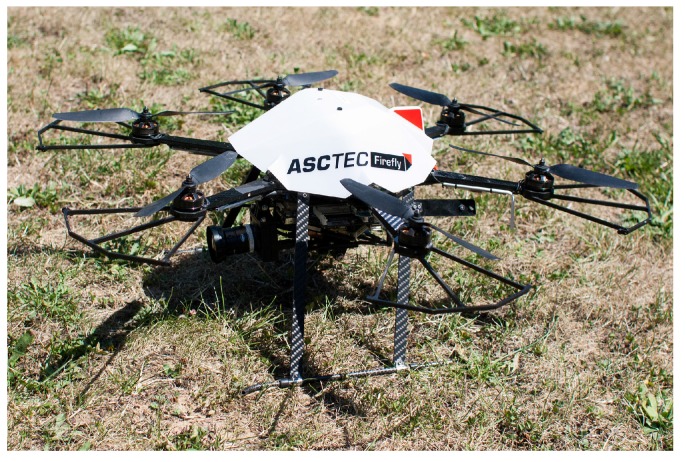
AscTec Firefly with the mounted uEye camera.

The UAV featured is an AscTec Firefly (as seen in Figure 12), with a processing computer on board. It runs ROS Indigo on Ubuntu 14.04. As a camera, a UEye UI-1240ML-C-HQ with a wide-angle lens has been mounted. The camera produces a 1280×1024 uncompressed eight-bit color image. It also has a global shutter to avoid rolling shutter issues, which are particularly problematic for UAV-mounted cameras. While it is possible to run the face detection on the UAV, this has been avoided to be able to run multiple tests on the same film material. For the recording process, the ROS rosbag package has been used. The footage is played back on an off-line computer with the same rosbag package, where the images are then processed by the face detection system. The processing computer also runs ROS Indigo in an Ubuntu 14.04 environment. The machine has an Intel Xeon(R) CPU E565 running 12 cores at 2.40 GHz with 5.8 GB of RAM.

### 6.4. Experiments

**Figure 13:**
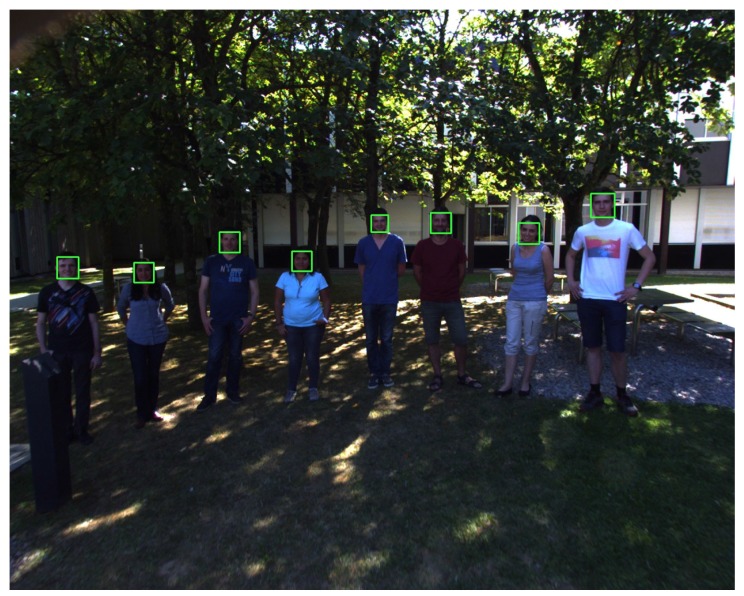
Shadow example: The detection is stable even during faster movement of the drone.

**Figure 14:**
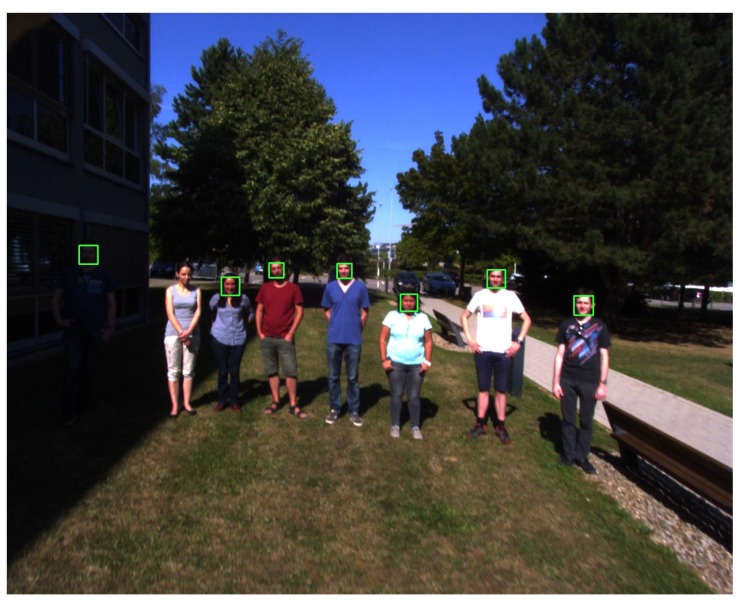
Direct sunlight example: note the detection of the person standing in the shadow.

**Figure 15:**
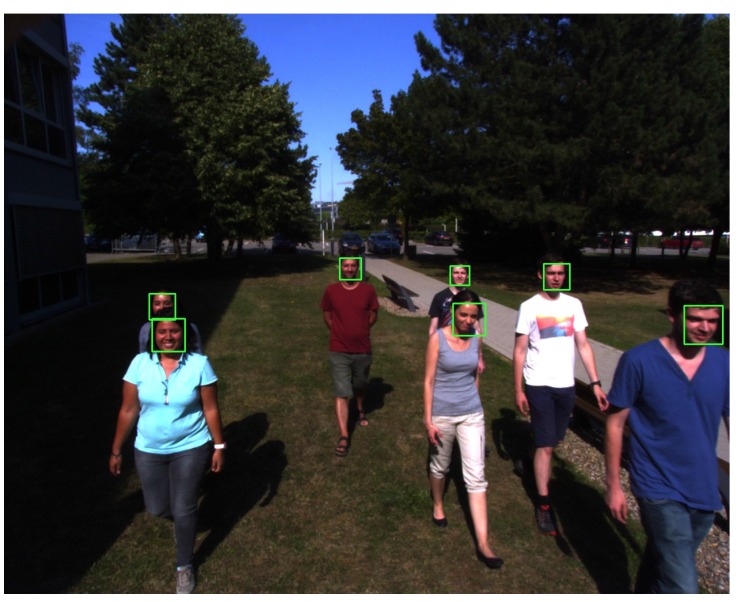
Fly-over example.

To ensure a realistic test environment, the drone was flown outside. The UAV was piloted with manual controls at a height of about 2.5–3.5 m. The direct sunlight provided additional difficulty, as there is much more contrast in the images. Multiple test situations where set up in order to prove the robustness of the system:

- Standing in the shadow: Faces are well exposed, and there are no harsh shadows. The face detection works well under these conditions, even during direction changes of the drone. One frame of this test is shown in Figure 13.
- Direct sunlight: When filming people standing in direct sunlight, the harsh shadows make the detection more difficult (Figure 14). In this case, the detection is not as consistent as in the previous test, but it still manages to detect all of the faces at some point. In Figure 14, it also shows how the system is able to detect a person who is standing in the shadow (left), even though the camera was not exposed for those lighting conditions, and it is difficult for humans eyes to even detect the body of this person.
- Fly-over: For the last experiment, the UAV was set to fly over a group of moving people. An example frame of this footage can be seen in Figure 15. Due to the close proximity to the subjects, this tests required the detection of a lot of faces of different sizes. The proximity also makes the motion blur on the subjects stronger. Because of the wide angle of the lens, lens distortion can also cause problems with closer subjects. In this case, the detection also works well, mainly because of the large size of the faces.

### 6.5. Calculation Speed

The calculation speed can fluctuate depending on the input images. On average, the detection speed has been consistent with a difference of only 0.03 frames per second over the three different test cases. While running on the previously-mentioned off-line machine, the system has been able to process an average of 4.258 frames per second.

### 6.6. Limitations

The current OpenCV implementation is very robust, even in difficult lighting conditions. One notable weakness of the face detection is people that are further away from the camera. Below a size of 10×10 pixels, the algorithm struggles to produce a good detection. This problem could, however, easily be fixed by using a higher resolution image sensor or by applying a lens with a narrower field of view. The detection also has difficulties with the orientation of the faces. Faces that are tilted sideways usually fall outside the detection range of the training model. Faces that are not front facing cannot be detected by the front-facing cascade model. OpenCV does, however, include a trained model for faces in profile view. In this case, only the frontal face model has been applied, since using a single model already requires much processing power. One should note here that people usually look at drones once they can hear them, which thereby leads to an easier detection. Lastly, partially-occluded faces are not always detected.

The videos related to some of the experiments presented in this section are available at [69].

## 7. Vision-Based Control Approach for Vehicle Following and Autonomous Landing

In previous sections, an animal tracking approach and a face detection technique using aerial images have been presented. However, for these to be promising in the fight against poachers, an autonomous control of the UAV is mandatory. This section presents a vision-based control approach to close the control loop using the result of an image processing algorithm as the input of the control. Our work includes vision-based control to follow suspicious vehicles (potential poachers) and to accomplish autonomous landing to recover the UAV after the end of a surveillance mission to recharge the batteries. This way, we are presenting a potential full surveillance mission in which the UAV takes off from a specific place and then follows GPS waypoints (these tasks are already integrated in most of the commercial available UAVs) to patrol a specific area of natural parks. If there is any animal or group of them detected during patrolling, it should be tracked to get information about the animal status to determine a potential poacher attack. In case people are found, the system has to detect the faces and store these data for security authorities (these two image processing algorithms were presented previously in this paper). Any potential vehicle should also be tracked. In this case, the UAV is able to follow the suspicious vehicle in a specific trajectory relatively to it. During the vehicle following task, the GPS position is shared with security authorities. Finally, the UAV has to come back to the closest (moving or static) base station and accomplish the autonomous landing to recharge its batteries and/or to be prepared for the next mission. In this section, we present the control approach to follow vehicles and to autonomously land on both static and moving bases.

### 7.1. Vision-Based Fuzzy Control System Approach

The presented control approach for UAVs is designed to use an onboard downwards looking camera and an inertial measurement unit (IMU). The information extracted from these two sensors is used to estimate the pose of the UAV in order to control it to follow vehicles and to land on the top part of ground vehicles or moving targets. The computer vision algorithm used is 3D estimations based on homographies. The homography detection is done with regards to a known target, which is an augmented reality (AR) code. The detection of this type of code is done with an ROS-implementation of the ArUco library [77]. The result of this algorithm is the pose estimation of the multi-copter with respect to the AR code. Multi-copters, as well as rotary wing platforms have a singularity with respect to fixed wings, which is the relation of the movement and the tilt of the thrust vector. It is not possible to move longitudinally (forward/backward) or laterally (left/right) without modifying the thrust vector. Because the camera is attached to the frame of the UAV, this movement significantly affects the estimations calculated by the vision algorithm. The estimation of the rotation of the UAV is retrieved from the gyroscope of the IMU. The subtraction of the roll and pitch rotations of the UAV from the image estimation is called de-rotation [78,79]. The relevant formulas are presented in Equation (17).

(17) $$\begin{array}{c} x^{\prime }=x-tan\left(\phi \right)\times z\\ y^{\prime }=y+tan\left(\theta \right)\times z\\ z^{\prime }=\sqrt{z^{2}-y^{\prime 2}} \end{array}$$

where x,y,z are the translation estimation on the x,y,z UAV axis, respectively, ϕ,θ are the roll and pitch rotations of the UAV, respectively, and $x^{\prime },y^{\prime },z^{\prime }$ are the resulting de-rotated translations of the x,y,z of the UAV, respectively.

**Figure 16:**
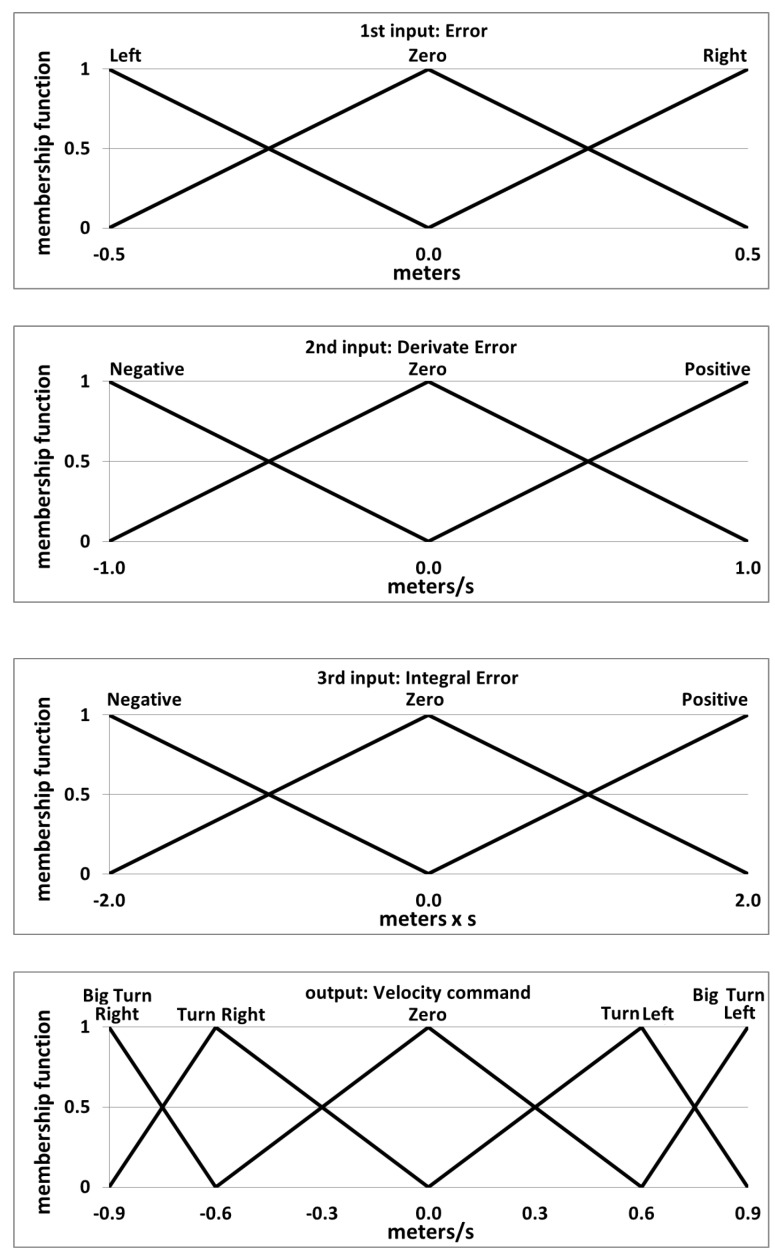
Final design of the variables of the fuzzy controller after the manual tuning process in the virtual environment (V-REP).

The de-rotated values of the pose estimation of the UAV are given as input to the control system. The control approach presented in this work consists of four controllers that are working in parallel, commanding the longitudinal, lateral and vertical velocities, as well as the orientation of the UAV. These four controllers are implemented as fuzzy logic PID-like controllers. The main reason for using this specific technique for the control loop is the way that this technique manages the uncertainty that is derived from the noisy data received from the vision-based detection algorithms and the IMU, as well as how it manages the high complexity of this type of non-linear robotics platform. Furthermore, the use of linguistic values by the fuzzy logic controllers simplifies the tuning process of the control system. The four fuzzy controllers were implemented using an in-house software called MOFS [80]. An initial configuration of the controllers was done based on heuristic information and was then subsequently tuned by using the Virtual Robotics Experimental Platform (V-REP) [81] and self-developed ROS modules. Detailed information about the tuning process can be found in [82]. In the present work, we use the same controller definition for the longitudinal, lateral and vertical velocity controllers, which is shown in Figure 16. The inputs are given in meters, and the output is calculated in meters × seconds. The orientation velocity controller gives the inputs in degrees, and the output is calculated in degrees × seconds. The control system has two different working states: In the first state, the UAV is set to follow the vehicles of a poacher while the height is predefined. The second state is used to recover the UAV for the next mission by landing it autonomously on top of the security entity’s vehicles.

In this work, an additional tuning phase has been included. It has been developed during experiments with a quadrotor tracking the target. Comparing to previous work [82], in this work, a weight value was assigned to each of the three inputs of each control variable, as well as to the output of each controller. The tuning process of these weights was done with the real quadrotor in real experiments. Table 1 shows the final values of the weight for all of the controllers after the tuning process.

**Table 1:** Tuned weight values for the four controllers.

Controller Weight	Lateral	Longitudinal	Vertical	Heading
Error	0.3	0.3	1.0	1.0
Derivative of the error	0.5	0.5	1.0	1.0
Integral of the error	0.1	0.1	1.0	1.0
Output	0.4	0.4	0.4	0.16

### 7.2. Experiments

**Figure 17:**
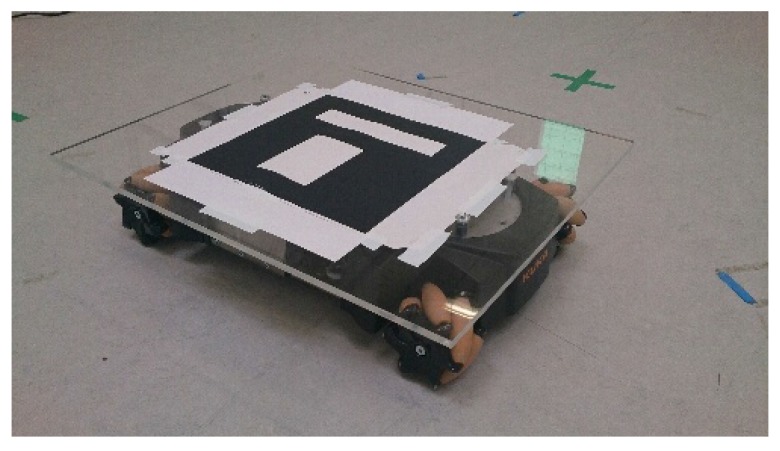
Youbot platform with the ArUco target.

The experiments have been done in the laboratory of the Automation & Robotics Research Group at the University of Luxembourg. The flight arena has a size of 6×5 m and a height of 5 m. The UAV used in the experiments is an AR.Drone v2.0 [83]. This platform is not equipped with an onboard computer to process the images. Therefore, the image processing and the control process are calculated remotely on a ground station. The delay of the WiFi communication affects the system by increasing the complexity of the non-linear system of the vision-based UAV control. A WiFi-router is used to reduce the maximum variations of the image rate. A Youbot mobile robot from KUKA [84] has been used as the target vehicle to follow, as well as for the autonomous landing task on a moving target. The top of this robot was equipped with a landing platform. The platform was covered with an ArUco code in order for it to be detected by the vision algorithm, as is shown in Figure 17. This ground platform was controlled randomly via ROS with a remote computer. The omnidirectional wheels of this ground robot allowed for the position of the tracked target to be modified in all directions. This freedom of movement and the height limitation increase the complexity of the following and landing tasks. This type of movement cannot be performed by normal vehicles.

Two different kinds of experiments were preformed. In the first experiment, the UAV had to follow the moving target from a fixed altitude of 3.5 m. In the second experiment, the UAV had to land on the moving ground platform. Table 2 shows the results of seven different experiments. The results are expressed with the root mean squared error (RMSE) for the evolution of the error for each controller. Depending on the controller, the RMSE is in meters (lateral, longitudinal and vertical velocity controller) or in degrees (heading velocity controller). The speed of the target platform was set to 0.5 m/s for the following Test #1, following Test #4 and the landing Test #3. For all other tests, the speed of the target platform was set to 0.3 m/s.

**Table 2:** Root mean square error for the lateral, longitudinal, vertical and heading velocity controllers for the autonomous following and landing on a moving target.

Controller	Lateral	Longitudinal	Vertical	Heading	time
Experiment	(RMSE, m)	(RMSE, m)	(RMSE, m)	(RMSE, Degrees)	(s)
Following #1	0.1702	0.1449	0.1254	10.3930	300
Following #2	0.0974	0.1071	0.1077	8.6512	146
Following #3	0.1301	0.1073	0.1248	5.2134	135
Following #4	0.1564	0.1101	0.0989	12.3173	144
Landing #1	0.1023	0.0.096	1.1634	4.5843	12
Landing #2	0.0751	0.0494	1.1776	3.5163	11
Landing #3	0.0969	0.0765	0.9145	4.6865	31

**Figure 18:**
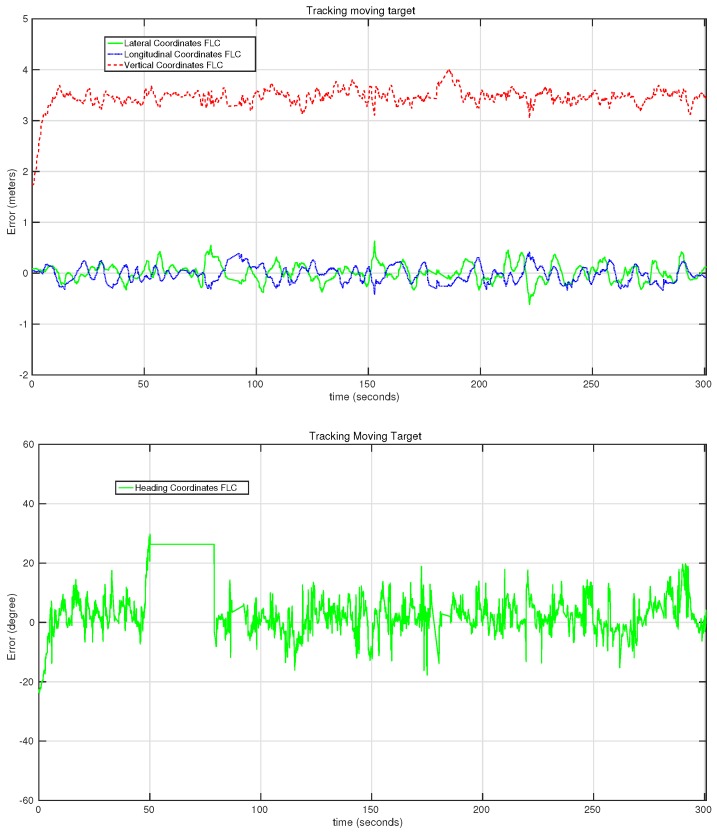
Evolution of the error of the lateral, longitudinal, vertical and heading controllers on the first moving target-following experiment.

The most important experiments are shown in the next graph. Figure 18 shows the behavior of the system in the first target-following experiment. While this was also the experiment with the longest duration, the RMSE error of the lateral and longitudinal is under 15 cm. An error in the estimation of the orientation of the target can be seen between the 50th and the 80th second of the test. This error, which was produced by the computer vision algorithm, did not affect the estimations for the lateral, longitudinal and vertical controllers.

**Figure 19:**
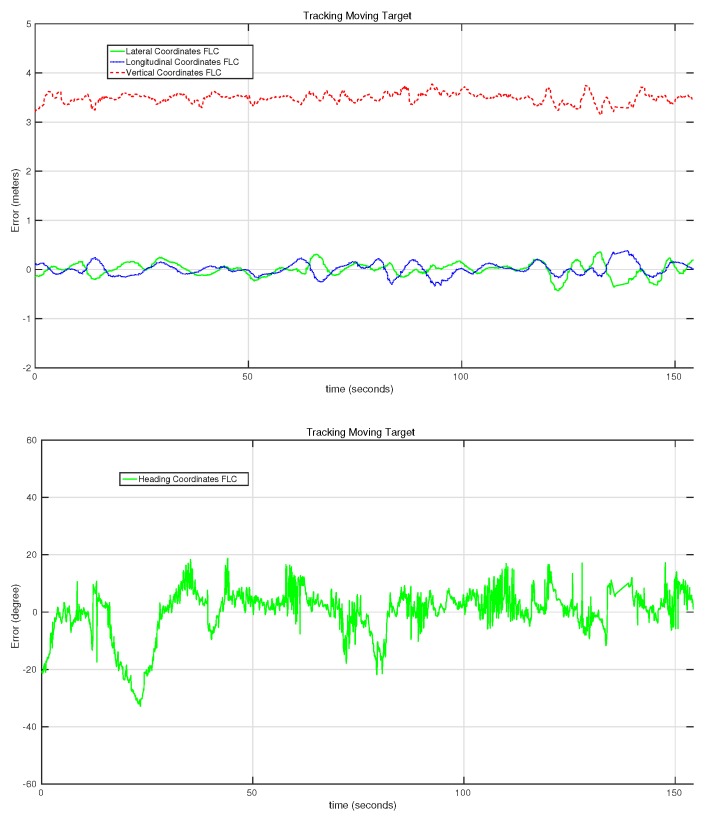
Evolution of the error of the lateral, longitudinal, vertical and heading controllers on the second moving target-following experiment.

Figure 19 shows the evolution of the error rate for all of the controllers in the second target-following experiment presented in Table 2. In this case, several orientation movements were applied to the target in order to evaluate the behavior of the heading controller in detail. This controller performs quickly, as can be seen in the two big changes at the first 50 s of the experiment. In this section of the test, the error reaches up to 35°, but the controller manages to reduce it in just a few seconds. During the other tests, more changes have been applied to the orientation of the target platform with similar performances of the heading controller. In this case, the RMSE of the heading controller was 8.6°.

**Figure 20:**
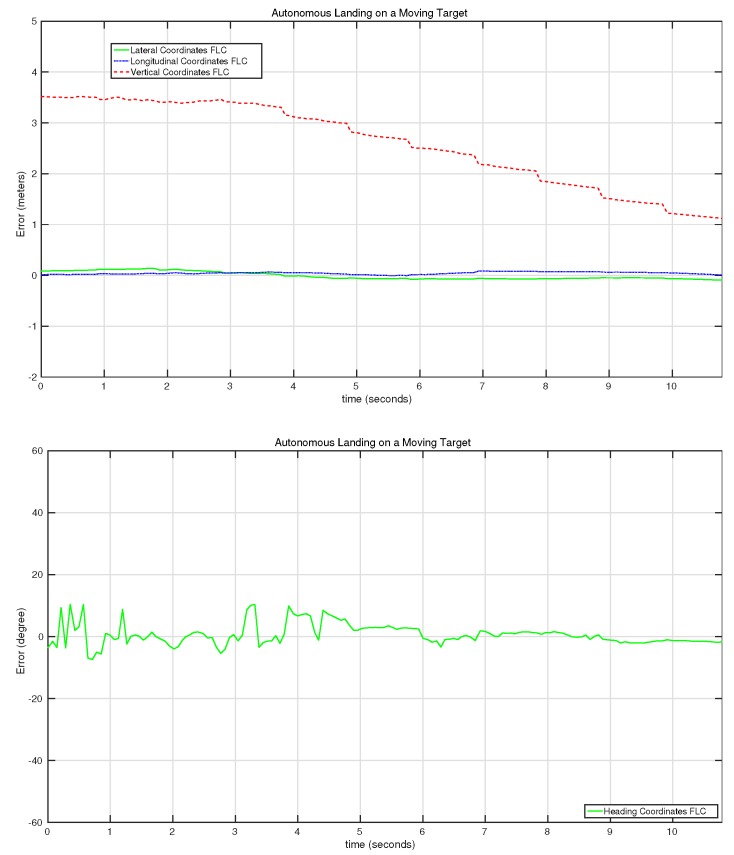
Evolution of the error of the lateral, longitudinal, vertical and heading controllers on the second autonomous landing on a moving target experiment.

Figure 20 shows the behavior of the controller for the second autonomous landing experiment. In this test, a gradual reduction of the altitude was performed by the UAV, reducing it from 3.5 m to 1 m in 8 s with an almost zero error for the lateral and longitudinal controllers. It has to be taken into account that the control system was set to reduce the vertical error up to 1 m, and then, a predefined landing command was sent to the UAV, which reduces the speed of the motors gradually.

**Figure 21:**
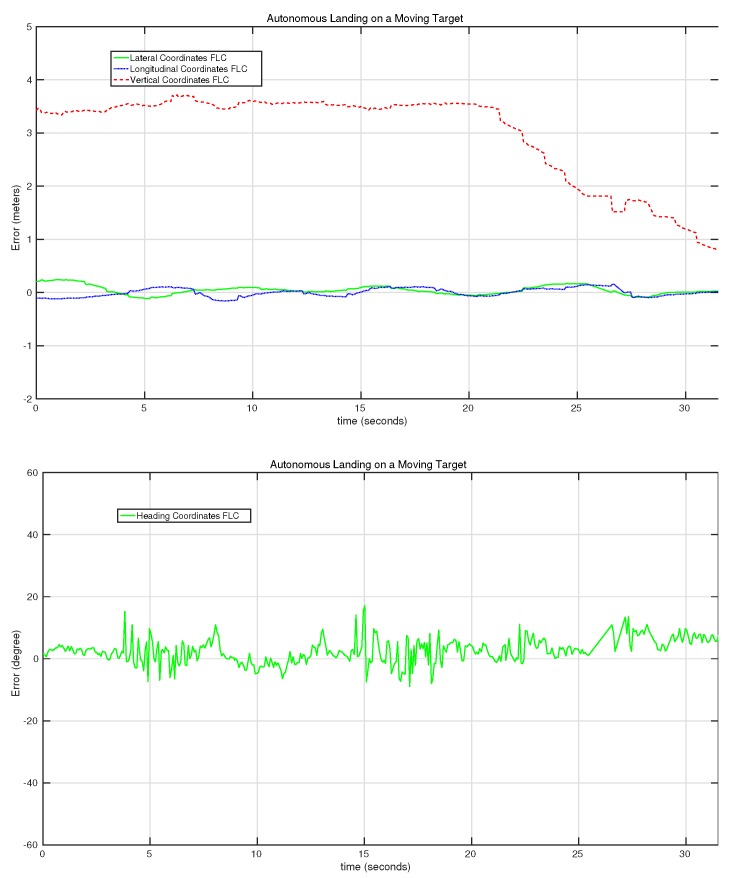
Evolution of the error of the lateral, longitudinal, vertical and heading controllers on the third autonomous landing on a moving target experiment.

Figure 21 shows the behavior of the control system for the third autonomous landing experiment. In this case, one can observe how the vertical controller pauses a couple of times for a few seconds in between the 25th and the 30th second of the test. This is because the vertical control system only sends commands when the errors of the heading, lateral and longitudinal controllers are smaller than some predefined values. This predefined behavior stabilized the landing process, reducing the potential loss of the target during the landing.

The videos related to some of the experiments presented in this section are available online [69].

## 8. Conclusions and Future Works

In this paper, the main engineering challenge and contribution is the integration of different techniques and algorithms to have a potential solution for setting up an autonomous surveillance system against anti-poaching activities. A surveillance mission consists of an autonomous take-off, detection and tracking of animals, detection and storage of poachers’ face data, an autonomous navigation of the UAV to follow suspicious vehicles and an autonomous landing for specific moving and/or static landing/recharging platforms.

An adaptive visual tracking algorithm was presented to track wildlife animals in their natural environment. The adaptive algorithm was tested with different aerial videos taken by quadcopters in Africa. In these videos, different animals in different natural environments were successfully tracked. A vision-based face detection algorithm is also presented to help with the detection, identification and creation of a database of poachers. The algorithm was successfully tested with aerial images captured with a quadrotor in different light conditions. The limitations of the camera configuration, which does not provide zoom optics, and the low resolution of the camera sensor reduced the distance at which people can be detected. An improvement of the camera configuration should improve the detecting distance for this algorithm. One of the remaining issues is that the detection is not always continuous. We are currently working on improving the detection with the help of image tracking algorithms. These algorithms are used to bridge the gap between successful detections. The mean shift algorithm was selected for this task, because it is not computationally intensive. Promising results in laboratory tests were achieved with the help of this technique, which still have to be tested in outdoor environments.

A vision-based control system approach was also presented in this paper in order to control a quadrotor to follow vehicles, as well as to land on a moving platform in order to achieve the recovery of the aircraft. This part of the work did not focus on developing a visual tracking system for the detection of the vehicles for the estimation of the relative position of the UAV. Instead, this issue was solved using augmented reality codes. In this case, we focused on the development of a vision-based control approach to command the UAV for these specific tasks. The control approach was done using fuzzy logic techniques, and the controllers were initially tuned in a simulated environment. A final tuning phase was conducted with the real quadrotor during real experiments in an indoor environment. A series of tests for the following and landing on a moving target was presented in this paper. The use of vision tracking algorithms is the next step to improve this vision-based control approach.
